# Appropriate Delivery Project: Impact of Simulation Training on the Increase in Vaginal Births in Hospitals in Brazil

**DOI:** 10.31744/einstein_journal/2024AO0783

**Published:** 2024-07-17

**Authors:** Mariana Santos Alecrim Molina, Eduardo Cordioli, Thomaz Bittencourt Couto, Joyce Kelly Silva Barreto, Rita de Cássia Sanchez

**Affiliations:** 1 Hospital Israelita Albert Einstein São Paulo SP Brazil Hospital Israelita Albert Einstein, São Paulo, SP, Brazil.

**Keywords:** Simulation training, Delivery, obstetric, Cesarean section, Brazil

## Abstract

**Objective:**

This study aimed to analyze the relationship between the participation of professionals in simulation-based training and an increase in the rate of vaginal deliveries.

**Methods:**

This retrospective observational study analyzed professionals’ participation in high-fidelity simulation training during the pilot phase of the Appropriate Delivery Project, spanning from May 21, 2015 to May 21, 2016, along with the rates of vaginal deliveries across various hospitals. Data for participation by nurses and physicians were examined using a gamma distribution model to discern the predictors influencing the changes in the percentage of vaginal births.

**Results:**

Data from 27 hospitals involved in the project were analyzed. A total of 339 healthcare professionals, including 147 nurses and 192 doctors, underwent the simulation-based training. During the pilot test, the percentage of vaginal births increased from 27.8% to 36.1%, which further increased to 39.8% in the post-intervention period, particularly when the participation rate of nurses exceeded the median.

**Conclusion:**

This study suggests that simulation-based training is a valuable strategy for achieving positive changes in obstetric practice, specifically an increase in the rate of vaginal births. These findings underscore the potential advantages of incorporating simulation training into improvement initiatives, as evidenced by the correlation between higher training adoption rates and substantial and sustained enhancements in vaginal birth rates.

## In Brief

The Appropriate Delivery Project aimed to reduce Brazil’s high rates of cesarean deliveries. Through multifaceted interventions including simulation-based training, it achieved a significant increase in vaginal deliveries. This study explores the vital role of simulation training in improving obstetric outcomes.

## Highlights

Simulation-based training is a valuable strategy for achieving positive changes in obstetric practice.

The rate of vaginal births increased after the intervention.

The findings underscore the potential advantages of incorporating simulation training into quality improvement initiatives.

Higher training adoption rates correlated with substantial and sustained enhancements in vaginal birth rates.

## Graphical abstract

**Figure:**
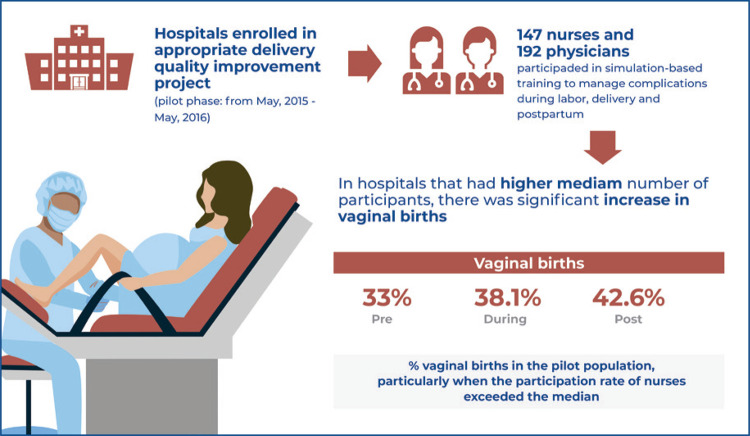

## INTRODUCTION

The rates of cesarean deliveries have increased significantly in most countries over the last three decades, and Brazil currently has the highest percentage of such deliveries worldwide. The overall percentage of cesarean deliveries across the private and public sectors in Brazil is 57%, which is much higher than the World Health Organization recommendation of an average cesarean delivery rate of 15% on the basis of scientific evidence.^(1)^ In the private sector alone, the cesarean delivery rate was 84.4% in 2015, with the rate being as high as 90% in some facilities.^(2)^

To change this scenario, a private hospital in Brazil and some national and international health agencies created the Appropriate Delivery Project (PPA), a collaborative project to improve the quality of obstetric care. The project was formulated in October 2014 and initiated in February 2015. This collaboration brought together 27 hospitals with high percentages of cesarean deliveries and employed several perspectives and multiple interventions to achieve a relative increase of 1.62 (95% confidence interval: 1.27-2.07, p<0.001) in the rate of vaginal deliveries over an 18-month period.^(3)^

During the pilot phase of this project, a series of measures encompassing the following four perspectives was implemented: hospital leadership, healthcare system (healthcare professionals and system structure), actions aimed at patients and families, and actions aimed at the information system in each hospital. Leaders and operational teams held face-to-face meetings in which the science of improvement, creation of new care models at birth, reinsertion of multidisciplinary teams in labor and delivery, visits to hospitals, assistance for physical restructuring of processes and protocols, and collection and monitoring of the rate of adverse events were taught. In addition, the following steps were implemented: meetings among the medical community on the precise indications for cesarean deliveries based on scientific evidence; creation of medical feedback models based on results, with reference to the collaborative averages; an improvement course in obstetric nursing for the reinsertion of these professionals in labor and delivery; and encouragement of interprofessional assistance. The measures also included actions to promote training of patients and family members about the advantages of physiological vaginal delivery, conversation circles, and visits to maternity hospitals to familiarize patients and their families to the physical spaces and the environment and to prepare them for labor and delivery. Finally, a theoretical-practical training program specifically aimed at resolving complications during labor, delivery, and the postpartum period was created at a simulation center to help general physicians, nurses, and obstetricians regain confidence in their technical capacity and teamwork.^(2)^

Simulation involves the creation of a situation or environment that allows people to experience the representation of a real event for practice, teaching, evaluation, or understanding of human systems or actions.^(4)^ This method can be used in interprofessional training in a cooperative educational activity by involving professionals from different health professions in activities in a simulated environment,^(5)^ and allows training of teams in skills that are known to increase the effectiveness and safety of healthcare teams, such as leadership and communication.^(6)^ Simulations have been widely used in obstetrics to teach undergraduate, graduate, and interprofessional teams.^(7-9)^ For the training of trained professionals, most courses described in the literature used simulation training in handling obstetric emergencies for physicians, nurses, and midwives, and the results showed a positive impact on confidence, knowledge, and skills.^(10,11)^ Nevertheless, studies proving the impact of simulations on clinical outcomes are lacking, as are studies aimed at the use of simulations in other areas of obstetric training.^(12)^ In a single study focusing on delivery techniques, Gosset et al. demonstrated that a training program with simulation focused on vaginal delivery, more specifically on the use of forceps by resident physicians, yielded a 26% reduction in severe lacerations and an increase in the correct indication of forceps.^(13)^ Therefore, while we were aware that the PPA included several actions and the impact of individual initiatives would be difficult to isolate, we hypothesized that training of professionals using high-fidelity simulations would show a direct correlation with increased rates of vaginal births in hospitals.

## OBJECTIVE

This study aimed to analyze the relationship between the participation of professionals from hospitals in simulation-based training and the reduction in the rate of cesarean deliveries.

## METHODS

This retrospective observational study was based on an analysis of the number of healthcare professionals who participated in high-fidelity simulation training during the pilot phase of the PPA from May 21, 2015, to May 21, 2016, and the vaginal deliveries performed at each hospital during the study period. The database for the study was obtained by collecting data on the number and training status of healthcare professionals, rates of vaginal and cesarean births, mortality and other complications at each institution as part of adherence to the appropriate birth program.

Each of the 27 hospitals involved in the pilot phase of the PPA that participated in the face-to-face learning sessions sent an average of 12 healthcare professionals, including physicians and nurses who provided obstetric care, to the national territory to undergo the training program; a total of 339 professionals underwent the training program, which was conducted at a simulation center in São Paulo, Brazil.

The educational objectives of the training program were as follows: train healthcare professionals in obstetric approaches with a focus on patient safety and promote the clinical reasoning necessary to manage possible obstetric complications; train professionals in directing proper delivery, particularly for monitoring labor and stimulation of the vaginal route; and practice the skills required to communicate bad news, encourage adherence to prenatal care and normal delivery, and assimilate concepts for conflict management.

The training was designed with the support of obstetrics experts and a realistic simulation center team and consisted of theoretical and practical modules. The duration of the theoretical module was 4 hours, and it covered the following topics: safety in obstetric practice, birth physiology and partograms, monitoring of labor, and assertive communication. The practical module included the use of patient simulators (SimMom - Laerdal^®^) during scenarios, static mannequins (Promp Birthing – Laerdal^®^; maternity simulation jacket - koken^®^) to train the professionals in practical skills (hands-on training), and professional actors in facilities similar to offices, Emergency Room and Obstetric Center, to simulate actual scenarios in which a certain procedure or behavior was to be employed, allowing better retention of information through a participatory environment. Approximately 38 students per class were divided into four groups to actively participate in tasks such as interpretation and discussion of a case with cardiotocography data, quantification and management of postpartum hemorrhage, pelvic birth maneuvers, forceps delivery, and extractor vacuum in the prolonged expulsive period, each lasting approximately 40 minutes. Training for the scenarios, on the other hand, was conducted in groups with a team of students who volunteered (two physicians and two nurses), lasted 10 minutes, and were transmitted to the other group participants, followed by a 25-minute debriefing, addressing the topics of birth with dystocia and companions in the room, shoulder dystocia, and adherence to the best indication (cesarean vs. vaginal).

For the analysis by hospital, a convenience sample was obtained from the 27 hospitals that participated in the project, had several healthcare providers who underwent the training program (n=339), and had data available on vaginal birth rates 13 months before the beginning of the pilot phase (April 2014 to April 2015) and 13 months after the end of the pilot phase (June 2016 to June 2017).

### Outcomes and measures

The participating hospitals uploaded monthly reports of data for indicators to a platform. This analysis was not intended to compare hospitals but to recognize improvements or sustained performance in each hospital throughout the duration of the project. To analyze the proportion of vaginal deliveries in the pilot study, we used data from April 2014 to June 2017 and divided it into three observation periods: April 2014 to April 2015 (Period 1), May 2015 to May 2016 (training period; Period 2), and June 2016 to June 2017 (Period 3).

We used the average number of deliveries per month for 13 months before the start of the pilot phase to represent the size of the hospital as a denominator to calculate the number of people trained. The median values of the total number of deliveries in the hospitals in February, March, and April 2015 were calculated and added to the database. Participation rates per hospital were calculated as follows: overall healthcare professional participation rate = number of trained professionals/every 100 deliveries; nurse participation rate = number of trained nurses/every 100 deliveries; and physician participation rate = number of trained physicians/every 100 deliveries. The variables observed per hospital were as follows: average number of deliveries per month over the 13-month period before the start of the pilot phase; vaginal birth rate over the 13-month period before the start of the pilot phase; vaginal birth rate over 13-month period after the training program; number of trained nurses; number of trained physicians; and total number of trained healthcare professionals.

Data for the qualitative variables were described as absolute and relative frequencies.^(14)^For the other variables, we used the gamma distribution because of data asymmetry. We fit the generalized estimating equation models using multiple approaches for each outcome.^(15)^In one of the multiple approaches, we considered the hospital class, number of nurses and physicians trained. The results are shown as estimated means by group and period, with 95% confidence intervals and p-values corrected using the sequential Bonferroni method. Analyses were performed using SPSS version 24, and the significance level was set at 5%.

### Ethics

This study was approved by the Ethics and Research Committee of *Hospital Israelita Albert Einstein*, CAAE: 24233019.0.0000.0071; # 3.780.954.

## RESULTS

### Participation data

We evaluated the data of 339 training participants, namely 147 nurses and 192 physicians, between May 2015 and May 2016. The median rates of trained professionals in relation to the number of monthly deliveries was 2.12 for nurses, 2.81 for physicians, and 5.41 for all healthcare professionals (Table 1).

**Table 1:** Number of trained professionals and participation rate by median number of deliveries

Hospital	Median number of deliveries*	Nurses	Physicians	Total	Nurses/100 deliveries	Physicians/100 deliveries	Professionals/100 deliveries
1	236	5	5	10	2.12	2.12	4.24
2	127	3	7	10	2.36	5.51	7.87
3	108	4	5	9	3.70	4.63	8.33
4	216	2	3	5	0.93	1.39	2.31
5	307	5	5	10	1.63	1.63	3.26
6	402	44	50	94	10.95	12.44	23.38
7	526	4	6	10	0.76	1.14	1.90
8	178	6	5	11	3.37	2.81	6.18
9	157	5	5	10	3.18	3.18	6.37
10	289	3	7	10	1.04	2.42	3.46
11	173	4	6	10	2.31	3.47	5.78
12	185	1	9	10	0.54	4.86	5.41
13	115	5	5	10	4.35	4.35	8.70
14	476	2	3	5	0.42	0.63	1.05
15	262	0	10	10	0.00	3.82	3.82
16	343	11	1	12	3.21	0.29	3.50
17	231	6	0	6	2.60	0.00	2.60
18	247	5	4	9	2.02	1.62	3.64
19	741	4	3	7	0.54	0.40	0.94
20	136	5	6	11	3.68	4.41	8.09
21	180	6	4	10	3.33	2.22	5.56
22	218	7	3	10	3.21	1.38	4.59
23	158	0	11	11	0.00	6.96	6.96
24	100	1	8	9	1.00	8.00	9.00
25	435	2	8	10	0.46	1.84	2.30
26	64	5	6	11	7.81	9.38	17.19
27	153	2	7	9	1.31	4.58	5.88

*During the 13-month pre-pilot period. Highlights in bold represent public hospitals.

### Vaginal deliveries in the pilot project

**Figure 1:**
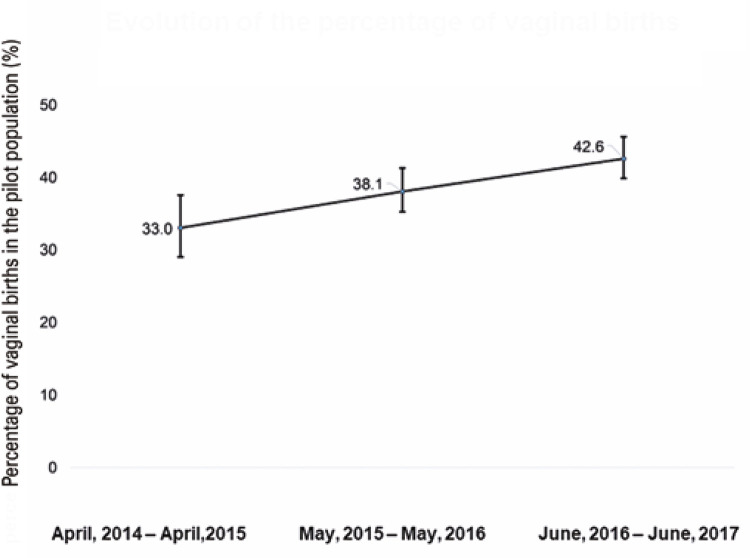
Evolution of the percentage of vaginal births in the pilot population

A total of 190, 335, and 295 observations were obtained in periods 1, 2, and 3, respectively. To assess the relationship between the rate of vaginal births in the pilot project and the other variables (Figure 1), we used a model with gamma distribution to investigate the following factors as possible predictors of changes in the percentage of vaginal births: the period, participation rate of nurses, participation rate of physicians, and the interaction between the period and other explanatory variables (Table 2).

**Table 2:** Description of hospital characteristics after categorization

Rate of trained nurses every 100 deliveries	
Median	2.12
≤2.12, n (%)	14 (51.9)
>2.12, n (%)	13 (48.1)
Rate of trained physicians every 100 deliveries	
Median	2.81
≤2.81, n (%)	14 (51.9)
>2.81, n (%)	13 (48.1)
Rate of trained professionals every 100 deliveries	
Median	5.41
≤5.41, n (%)	14 (51.9)
>5.41, n (%)	13 (48.1)

n=27.

To include the participation rates of nurses and physicians in the model, we created categories for the three quantitative variables from the medians observed in the sample (Table 3).

**Table 3:** Comparisons between periods for the percentage of vaginal deliveries in the pilot population

	Period 1 %	Period 2 %	Period 3 %	p value Period 2 × Period 1	p value Period 3 × Period 1	p value Period 3 × Period 2
Global	33.0 (29.0; 37.6)	38.1 (35.2; 41.3)	42.6 (39.8; 45.6)	0.064	<0.001	0.004
Hospital class						
Public	49.0 (36.0; 66.8)	45.7 (41.2; 50.6)	49.8 (45.1; 55.0)	>0.99	>0.99	0.541
Private	22.3 (18.8; 26.3)	31.8 (28.7; 35.3)	36.5 (33.3; 39.9)	<0.001	<0.001	<0.001
Rate of trained nurses per 100 deliveries						
≤2.12	39.2 (28.9; 53.2)	40.2 (36.1; 44.8)	45.7 (41.2; 50.6)	0.855	0.522	0.014
>2.12	27.8 (24.8; 31.2)	36.1 (31.4; 41.5)	39.8 (35.6; 44.5)	<0.001	<0.001	0.046
Rate of trained physicians every 100 deliveries						
≤2.81	30.1 (24.2; 37.6)	35.0 (31.4; 39.0)	41.6 (37.3; 46.4)	0.179	0.006	0.001
>2.81	36.2 (31.1; 42.1)	41.5 (36.6; 47.2)	43.7 (39.2; 48.6)	0.189	0.020	0.227
Rate of trained professionals every 100 deliveries						
≤5.41	36.2 (27.9; 46.9)	38.0 (33.1; 43.5)	44.7 (39.5; 50.5)	0.649	0.075	0.005
>5.41	30.5 (26.0; 35.8)	39.5 (34.4; 45.4)	42.2 (37.0; 48.2)	<0.001	<0.001	0.157

### Adverse event rate

In the assessment of adverse events, a total of 15, 236, and 135 observations were recorded in periods 1, 2, and 3, respectively (Table 4).

**Table 4:** Comparisons of the rate of adverse events between periods

	Period 1 (%)	Period 2 (%)	Period 3 (%)	p value Period 2 × Period 1	p value Period 3 × Period 1	p value Period 3 × Period 2
Global	57.2 (48.7; 67.1)	48.3 (41.2; 56.7)	53.9 (39.8; 73.1)	0.099	0.674	0.629
Hospital class						
Public	--	45.2 (34.7; 58.9)	60.7 (35.5; 103.9)	--	--	0.222
Private	57.2 (48.7; 67.1)	51.7 (42.3; 63.2)	47.9 (37.9; 60.4)	<0.001	0.068	0.426

## DISCUSSION

This retrospective study analyzed the clinical outcomes of hospitals participating in the PPA and the participation of professionals from these hospitals in the training program and found a direct correlation between the participation of physicians and nurses in simulation-based training and the increased rate of vaginal deliveries.

The National Supplementary Health Agency has been working for more than a decade to reduce the rate of cesarean deliveries in Brazil through events to discuss the topic, rewarding Qualification Program points to operators with a lower proportion of cesarean deliveries, developing educational materials, publishing regulations, funding research on the causes and consequences of cesarean deliveries in the supplementary sector, and making available newsletters on cesarean delivery rates, guidelines, and initiatives in favor of vaginal birth. However, these actions proved to be insufficient to substantially reduce the predominance of cesarean deliveries in the supplementary health sector during the period from 2004 to 2014.^(2)^

One of the ways to evaluate the effectiveness of a training program was described by Kirkpatrick et al. as follows: level 1, Reaction: participants’ perceived value in participating in the training; level 2, Learning: knowledge, skill, or attitude gained as a result of training; level 3, Behavior: gain in knowledge, skills, and attitudes transferred to the clinical environment; level 4, Result: improved outcomes achieved with training.^(16)^ The study indicated that the simulation-based training yielded level 4 results, that is, the presence of professionals in the simulation was related to the desired clinical outcome: an increase in vaginal deliveries. Although the simulations were part of a project with several action points to reduce cesarean deliveries, to the best of our knowledge, no related studies have used this teaching methodology as a strategy. Another important point is that the results obtained in the pilot year were sustained in the following year, which demonstrated a change in the organizational culture and model of sustained care in institutions.

One of the limitations of our study is the impossibility of attributing both the change and its sustained nature exclusively to simulation training, since several other initiatives took place simultaneously. In fact, simulation is likely to show the maximum effect when it is integrated within a curriculum, not when it is performed in isolation.^(17)^ Thus, the findings of this study provide important evidence for hospital managers to include simulation-based training as a valid strategy in improvement projects in obstetric practice.

Some limitations of this study include its retrospective design, which limited the interpretation of the results because of the inherent selection bias caused by necessarily selecting data from the most participative professionals. However, the large number of hospitals and professionals evaluated helped minimize this bias. The use of strategies other than simulation-based training as well as the different characteristics of the trained hospitals prevented the establishment of a linear relationship between training and results. However, the fact that we found an association between a greater number of trained professionals and better clinical outcomes in hospitals demonstrates the substantial potential benefits of training. Finally, we were unable to establish the effect of training on complication rates related to obstetric care. This was probably because notification of these events was very poor before the project began, preventing any changes in these indicators from being detected.

A possible follow-up to this study would be to measure the impact of simulation-based training in the years following the PPA, as it continues with an even greater number of Brazilian hospitals. Because simulation training involves high costs of environment, equipment, and teaching dedication, the calculation of training costs against cost gains with the reduction in unfavorable clinical outcomes would also be useful in guiding the management of future improvement projects.

## CONCLUSION

The inclusion of simulation-based training for obstetric healthcare professionals was associated with an increase in the number of vaginal deliveries in Brazilian hospitals.
